# Delays in obstetric care increase the risk of neonatal near-miss morbidity events and death: a case-control study

**DOI:** 10.1186/s12884-020-03128-y

**Published:** 2020-07-29

**Authors:** Ocilia Maria Costa Carvalho, Antônio Brazil Viana Junior, Matheus Costa Carvalho Augusto, Álvaro Jorge Madeiro Leite, Rivianny Arrais Nobre, Olivia Andrea Alencar Costa Bessa, Eveline Campos Monteiro de Castro, Fernanda Nogueira Barbosa Lopes, Francisco Herlânio Costa Carvalho

**Affiliations:** 1 Departamento de Saúde Comunitária / Universidade Federal do Ceará. Fortaleza, Fortaleza, CE Brazil; 2 Departamento de Saúde Materno Infantil / Universidade, Fortaleza, CE Brazil; 3 Universidade de Fortaleza/ UNIFOR, Fortaleza, CE Brazil; 4 Departamento de Saúde da Mulher, da Criança e do Adolescente UFC, Fortaleza, Brazil; 5 Maternidade Escola Assis Chateaubriand, Fortaleza, CE Brazil

**Keywords:** Near-miss, Healthcare, Comprehensive healthcare, Infant, Mortality

## Abstract

**Background:**

To evaluate the association between delays in obstetric care and neonatal near-miss mortality events and death in a public maternity referral center.

**Methods:**

This case-control study enrolled 142 neonates, meeting the near-miss criteria of 5-min Apgar < 7, weight < 1500 g, gestational age < 32 weeks, and use of mechanical ventilation or congenital malformation, as well as 284 controls (without the near-miss criteria), at a ratio of 1:2. After follow-up, the following outcomes were reclassified: survival of the neonatal period without the near-miss criteria (true “controls”), “near-miss,” and “neonatal death.” Maternal sociodemographic characteristics, prenatal care, and pregnancy resolution were evaluated. Pearson’s chi-square and Fisher’s exact tests were used. Simple logistic regression was performed to determine the association between the three delay factors with near-miss outcomes and/or neonatal death. The variables that had maintained values of *p* < 0.05 were subjected to multinomial logistic regression.

**Results:**

Comparisons revealed the following associations: for controls and near-miss events, delayed access to health services due to a lack of specialized services (odds ratio [OR], 3.0; 95% confidence interval [CI], 1.8–5.1) and inappropriate conduct with the patient (OR, 12.1; 95% CI, 1.3–108.7); for controls and death, absent or inadequate prenatal care (OR, 3.3; 95% CI, 1.6–7.1) and delayed access to health services due to a lack of specialized services (OR, 2.5; 95% CI, 1.1–5.6); and for near-miss events and death, absent or inadequate prenatal care (OR, 2.2; 95% CI, 1.0–5.0). Logistic regression for the combined outcome (near-miss plus neonatal deaths) revealed absent or inadequate prenatal care (OR, 1.9; 95% CI, 1.2–2.8), lack of specialized services (OR, 2.8; 95% CI, 1.7–4.5), and improper conduct with the patient (OR, 10.6; 95% CI, 1.2–91.8).

**Conclusions:**

The delays in obstetric care associated with the presence of near-miss and/or neonatal death included absent or inadequate prenatal care, delayed access to health services due to a lack of specialized services, and inappropriate conduct with the patient.

## Background

Neonatal morbimortality is used as a parameter to evaluate the quality of healthcare during the prenatal and childbirth periods. It also helps evaluate population healthcare and socioeconomic development levels [1, 2].

Neonatal near-miss morbidity is defined as the near death of a newborn due to a serious complication during the first 28 days of life. Studies have shown that the number of survivors affected by such morbidities is two to six times higher than the number of neonates who died [3].

The main components of infant mortality are concentrated in the neonatal period. In 2016, approximately 5.6 million children died worldwide, almost half (46%) within the first 28 days of life [4].

The main causes of mortality in the neonatal period are infectious diseases and neonatal complications. In Brazil, 39% of these deaths are associated with the care provided to pregnant women and 14% are directly associated with delivery [5, 6]. In this context, avoidable causes are protagonists and relevant obstetric complications. Factors involving neonatal morbimortality are inseparable from maternal health problems, being influenced by prenatal, delivery, and postnatal continuous care in the health system [3, 6].

Neonatal mortality rates have decreased worldwide. Brazil has followed this trend by providing important measures, such as a mother and baby healthcare program with direct actions related to pregnancy, delivery, and birth [7, 8]. Despite this advance, statistics are still alarming and demand urgent action.

In the 1990s, Thaddeus and Maine created an evaluation model to study maternal deaths since they were concerned about how pregnant women arrived at the hospital and how it could affect their health and the newborns. This is known as the “three delays” model, which relates mortality to a series of delays in obstetric care that prevent women from accessing skilled and effective care at various healthcare levels. These delays may be associated with personal, sociocultural, and institutional causes [9, 10].

Recent studies have replicated this methodology to understand maternal mortality and its effect on newborns. From this perspective, this study aimed to evaluate the association between delays in obstetric care and the presence of near-miss events and neonatal death.

## Methods

This case-control study was designed to identify factors associated with near-miss neonatal morbidity conducted between January and December 2017 at the Assis Chateaubriand Teaching Maternity (Federal University of Ceará), a referral hospital for high-risk pregnancies in Northeast Brazil. Assis Chateaubriand Teaching Maternity provides healthcare services to predominantly low-income population. Approximately 5000 infants are delivered each year at the hospital. It offers Fetal Medicine services and has a Maternal and Neonatal Intensive Care Unit. Of the total 4929 deliveries, 392 newborns had at least one of the pre-established neonatal near-miss criteria in 2017. A total of 426 mothers, including cases and controls, agreed to respond to a specific interview about their experiences during pregnancy and delivery.

The study sample size included 142 cases initially selected using the neonatal near-miss criteria and 284 controls in a 1:2 ratio. If a patient would die, the case would be reclassified as death. The inclusion criteria was neonatal near-miss according to Silva et al. (2014) [7]: 5-min Apgar score < 7, weight < 1500 g, gestational age < 32 weeks, use of mechanical ventilation, or presence of congenital malformation. The control group included newborns who did not meet the eligibility criteria born immediately before or after a selected case.

The exclusion criteria for cases included the following: newborns whose information could not be obtained from medical records or by interview with family members; deliveries that were considered abortions (< 20 gestational weeks and weight < 500 g); newborns with malformations or chromosomal syndromes considered lethal; and deliveries that occurred outside the hospital environment (e.g., in an ambulance). For controls, the exclusion criteria included the following: newborns whose information could not be obtained from medical records or through interviews with family members and deliveries that occurred outside the hospital environment.

The newborns were followed-up during the neonatal period. After follow-up, cases and controls were reclassified by outcome: survival to neonatal period without near-miss criteria (true controls), near-miss, and neonatal death.

Cases and controls were identified through a survey of the birth records of the neonatology unit of the maternity ward. A standardized questionnaire was used to collect socioeconomic data, maternal characteristics, prenatal and newborn information, complications during pregnancy and childbirth, and the mother’s previous health.

All mothers provided written informed consent form and were interviewed in the first 24 h after delivery.

The analysis of the three delays followed the criteria established by the World Health Organization [11]. The first delay was in deciding to seek appropriate medical help (family/patient) as follows. **Refusal of care** was identified when the mother did not seek prenatal care despite not desiring the pregnancy, did not attend appointments due to not liking the medical and nursing staff, the health unit being too far from home, or caring for another child, did not undergo tests due to lack of time, or hospital evasion; **delay in seeking care** was caused due to a change of address or other personal issues; and **was unaware of the signs** was when the mother did not know she was pregnant or sought care only when she was in a more severe clinical state.

The second delay was related to reaching an appropriate health service as follows. **Absent or inadequate prenatal care** occurred when the mother started prenatal care after the 12th gestational week, was unavailable to undergo all recommended tests, had fewer than six consultations, had no access to prenatal care due to a lack of staff or difficulties reaching high-risk prenatal care—the patient recognizes the need to start prenatal care, but cannot attend it because of difficulties beyond her control; **difficult access due to a lack of beds,** was when the pregnant woman went to more than one maternity ward seeking care in urgent/emergency situations; **difficult access due to a lack of specialized care** occurred when no hospital specialized in high-risk care or no specialized staff—data confirmed by transfer reports from the ambulance service; **difficult communication between hospital and control center** occurred when the patient arrived at the hospital with no guarantee of consultation or hospitalization; **transportation problems** were defined as the lack of an ambulance—when the patient was referred from another health unit, but arrived by car or taxi; and **difficult geographical access to health services** was experienced when the patient could not reach the hospital for delivery.

The third delay regarded receiving good healthcare at the health unit included the following: **delay in diagnosis, delay in transferring/referring the case, improper conduct with a patient, lack of medication or blood products, and delay in starting treatment.** For example, delay in recognizing signs of severity in cases of pre-eclampsia and, therefore, need to initiate magnesium sulfate. The medical records were evaluated by two high-risk specialist obstetricians who were blinded to the group assignments. There was no disagreement regarding any of the evaluated cases. The assessment regarding the third delay was performed specifically to define it as a parameter in this study protocol. There is no audit service at the institution. All cases of maternal, fetal, and neonatal deaths are evaluated by the Death Committee, which included medical professionals, nurses, and epidemiologists. This committee seeks to determine whether death is avoidable, but does not directly determine the delays in healthcare.

The Pearson’s chi-square and Fisher’s exact tests were used in this study. Simple logistic regression was used to determine the strength of association of the three delay factors with near-miss and death outcomes for variables with *p* values < 0.05. Multinomial logistic regression included variables with p values < 0.05 in simple regression [12]. Odds ratios (OR) were calculated with respective confidence intervals (95% CI). Statistical tests did not consider values without information. SPSS version 24.0 for Windows® and R version 3.31 software were used for the statistical analysis. The data were compiled using Excel® software (2010). G*Power 3.1.9.2 software determined that the sample power was 98.3% [13]. The sample power was calculated a posteriori using the following input parameters: z tests - Logistic regression Options: Large sample z-Test, Demidenko (2007) with var. corr Analysis: Post hoc: Compute achieved power Input: Tail(s) = Two Odds ratio = 2.27 Pr(Y = 1|X = 1) H0 = 0.382 α err prob. = 0.05 Total sample size = 426 R^2^ other X = 0.038 X distribution = Binomial X parm π = 0.46 Output: Critical z = 1.9599640 Power (1-β err prob) = 0.9832087.

This study was approved by our institution’s research ethics committee (no. 1,869,528) in compliance with Resolution 466/12 of the National Health Council on Research with Human Beings.

## Results

Of the 4929 births, 392 newborns met at least one of the neonatal near-miss criteria. The sample included 142 cases and 284 controls selected using a specific interview with the three delay variables. The newborns were followed up during the neonatal period until hospital discharge or the 28th day of life (whichever occurred first). After follow-up, cases and controls were reclassified according to the outcomes: survival of the neonatal period without near-miss criteria (true controls), near-miss, and neonatal death.

Of the variables studied, the mother’s origin and occupation were associated with delays and neonatal near-miss and death. Neonates of *pardo* mothers with higher education levels (≥ 8 years), who were living with a partner in the capital, who do not work outside the home, who are aged 19–34 years, and with income less than the minimum were more prevalent (Table 1).

**Table 1:** Sociodemographic characteristics of the mothers by group and deaths. Fortaleza CE, 2017

Variable	Control n (%)		NNM n (%)		Deaths n (%)		P*
**Sociodemographic characteristic**							
**Age range**							0.381
≤18 years	38	(13.4)	13	(12)	4	(11.8)	
19–34 years	206	(72.5)	72	(66.7)	22	(64.7)	
> 34 years	40	(14.1)	23	(21.3)	8	(23.5)	
**Education**							0.980
< 8 years	79	(27.8)	30	(27.8)	10	(29.4)	
≥8 years	205	(72.2)	78	(72.2)	24	(70.6)	
**Marital status**							0.449
With partner	238	(83.8)	85	(78.7)	29	(85.3)	
Without partner	46	(16.2)	23	(21.3)	5	(14.7)	
**Race**							0.461
White	60	(21.2)	26	(24.1)	10	(29.4)	
Black	37	(13)	10	(9.3)	6	(17.6)	
*Pardo*	187	(65.8)	72	(66.7)	18	(52.9)	
**Origin**							**0.016**
Capital/outskirts	250	(88)	86	(79.6)	24	(70.6)	
Countryside	34	(12)	22	(20.4)	9	(26.5)	
No information	0	(0)	0	(0)	1	(2.9)	
**Occupation**							**0.024**
Work	87	(30.6)	49	(45.4)	12	(35.3)	
Do not work	197	(69.4)	59	(54.6)	22	(64.7)	
**Income**							0.348
More than minimum	67	(23.6)	24	(22.2)	6	(17.6)	
Less than minimum	135	(47.5)	50	(46.3)	14	(41.2)	
No information	82	(28.9)	34	(31.5)	14	(41.2)	
**Number of pregnancies**							0.511
1–2	172	(60.5)	69	(63.9)	17	(50)	
3–4	95	(33.5)	31	(28.7)	13	(38.2)	
5 and more	17	(6)	8	(7.4)	4	(11.8)	

*NNM* Neonatal near-miss *Pearson’s Chi-square test.

Of the mothers studied, 37.8% had a premature delivery and 62.9% had a cesarean section. Of the neonatal near-miss cases, 30.6% were classified as high-risk pregnancies and required specialized services. The mortality rate was 26.5%. Regarding the number of consultations, 50.9% had less than six; of that group, 64.7% of the infants died (Table 2).

**Table 2:** Characteristics of prenatal care and pregnancy outcomes in controls, NNM cases, and deaths. Fortaleza CE, 2017

Variable	Control n (%)		NNM n (%)		Deaths n (%)		P
**No. of prenatal consultations**							0.001*
< 6	110	(38.7)	55	(50.9)	22	(64.7)	
≥6	170	(59.9)	50	(46.3)	10	(29.4)	
No information	4	(1.4)	3	(2.8)	2	(5.9)	
**Prenatal service**							0.037*
Primary healthcare	212	(74.6)	70	(64.8)	21	(61.8)	
Specialized care required (high-risk)	60	(21.1)	33	(30.6)	9	(26.5)	
Private clinic	3	(1.1)	2	(1.9)	1	(3.2)	
No information	9	(3.2)	3	(2.8)	3	(8.8)	
**Orientation on place of delivery**							0.912*
Yes	186	(65.5)	72	(66.7)	21	(61.8)	
No	94	(33.6)	33	(31.4)	11	(34.4)	
No information	4	(1.4)	3	(2.8)	2	(5.9)	
**Number of maternity hospitals visited**							0.170*
≥3	12	(4.2)	2	(1.8)	1	(2.9)	
2	82	(28.9)	45	(41.7)	12	(35.3)	
1	187	(65.8)	61	(56.5)	21	(61.8)	
No information	3	(1.1)	0	(0.0)	0	(0.0)	
**If transferred**							< 0.001*
Regulated	21	(7.4)	18	(16.7)	8	(23.5)	
Referred	45	(15.8)	38	(35.2)	7	(20.6)	
Own discretion	215	(75.7)	51	(47.2)	19	(55.9)	
No information	3	(1.1)	1	(0.9)	0	(0.0)	
**Type of delivery**							< 0.001**
Cesarean section	160	(56.3)	86	(79.6)	22	(64.7)	
Forceps	2	(0.7)	0	(0)	0	(0)	
Vaginal	122	(43)	22	(20.4)	12	(35.3)	

*Pearson’s chi-square test; **Fisher’s exact test

Among the three delay variables, absent or inadequate prenatal care and difficult access due to a lack of specialized services (second delay) and improper conduct with patient (third delay) showed statistical significance with *p* < 0.05 (Table 3).

**Table 3:** The effect of the three delays by group and deaths. Fortaleza CE, 2017

Delay type	Control	(%)	NNM	(%)	Death	(%)	P
**1. DELAY IN DECIDING TO SEEK APPROPRIATE MEDICAL HELP (FAMILY/PATIENT)**							
**Delay seeking care**							0.153*
Yes	50	(17.6)	12	(11.1)	8	(23.5)	
No	234	(82.4)	96	(88.9)	26	(76.5)	
**Unaware of pregnancy**							0.583**
Yes	10	(3.5)	5	(4.6)	0	(0)	
No	274	(96.5)	103	(95.4)	34	(100)	
**Refusal of care**							0.317*
Yes	33	(11.6)	15	(13.9)	7	(20.6)	
No	251	(88.4)	93	(86.1)	27	(79.4)	
**2. DELAY IN REACHING APPROPRIATE HEALTH SERVICE**							
**Absent/inadequate prenatal care**							0.001*
Yes	90	(31.7)	45	(41.7)	21	(61.8)	
No	194	(68.3)	63	(58.3)	13	(38.2)	
**Lacking specialized care**							< 0.001*
Yes	43	(15.1)	39	(36.1)	11	(32.4)	
No	241	(84.9)	69	(63.9)	23	(67.6)	
**Lacking beds**							0.086*
Yes	27	(9.5)	4	(3.7)	1	(2.9)	
No	257	(90.5)	104	(96.3)	33	(97.1)	
**3. DELAY IN RECEIVING CARE AT THE HEALTH UNIT**							
**Delay in diagnosis**							0.502**
Yes	2	(0.7)	2	(1.9)	0	(0)	
No	282	(99.3)	106	(98.1)	34	(100)	
**Delay in case transfer/referral**							0.198*
Yes	8	(2.8)	4	(3.7)	3	(8.8)	
No	276	(97.2)	104	(96.3)	31	(91.2)	
**Improper conduct with patient**							0.007*
Yes	1	(0.4)	5	(4.6)	1	(2.9)	
No	283	(99.6)	103	(95.4)	33	(97.1)	
**Delay in initiating treatment**							0.256*
Yes	5	(1.8)	2	(1.9)	2	(5.9)	
No	279	(98.2)	106	(98.1)	32	(94.1)	

*Pearson’s chi-square test; **Fisher’s exact test

Multinomial logistic regression was used to determine the association between the three delay risk factors and neonatal near-miss and deaths. The variables with values of *p* < 0.05 analyzed with multinomial logistic regression were absent or inadequate prenatal care, delayed access to health services due to a lack of specialized care, and improper conduct with the patient. Absent or inadequate prenatal care was significant in the intergroup comparisons between control and death and near-miss and death; delayed access to healthcare due to a lack of specialized services showed a significant intergroup association between control and death, and improper conduct with the patient also showed a significant intergroup association (Table 4).

**Table 4:** Logistic regression of the three delays by group and death outcomes. Fortaleza CE, 2017 2017

	Control × Near-miss		Control × Death		Near-miss × Death	
Variable	OR (95% CI) Gross	OR (95% CI) Adjusted	OR (95% CI) Gross	OR (95% CI) Adjusted	OR (95% CI) Gross	OR (95% CI) Adjusted
Absent or inadequate prenatal care	1.5 (0.9–2.4)	1.5 (0.9–2.4)	1.5 (0.9–2.4)	**3.4** **(1.6**–**7.1)**	**3.5** **(1.7–7.3)**	**2.3** **(1.03**–**5.0)**
Delay due to lack of specialized service	**3.2** **(1.9**–**5.3)**	**3.1** **(1.8**–**5.1)**	**2.7** **(1.2**–**5.9)**	**2.5** **(1.1**–**5.7)**	0.8 (0.4–1.9)	0.8 (0.4–1.9)
Improper conduct with the patient	**13.9** **(1.6**–**120.9)**	**12.1** **(1.4**–**108.8)**	8.7 (0.5–142.5)	7.7 (0.5–132.8)	0.6 (0.1–5.5)	0.7 (0.1–5.1)

*OR* Odds ratio; *CI* Confidence interval.

Univariate and multinomial logistic regression analyses considering a combined outcome (near-miss and neonatal deaths) are shown in Tables 5 and 6, respectively.

**Table 5:** Effect of the three delays considering combined outcome (near-miss and neonatal deaths). Fortaleza CE, 2017

Delay type	Control	(%)	NNM + Death	(%)	P
**1. DELAY IN DECIDING TO SEEK APPROPRIATE MEDICAL HELP (FAMILY/PATIENT)**					
**Delay seeking care**					0.355*
Yes	50	(17.6)	20	(14.1)	
No	234	(82.4)	122	(85.9)	
**Unaware of pregnancy**					> 0.999 *
Yes	10	(3.5)	5	(3.5)	
No	274	(96.5)	137	(96.7)	
**Refusal of care**					0.216*
Yes	33	(11.6)	22	(12.9)	
No	251	(88.4)	120	(87.1)	
**2. DELAY IN REACHING APPROPRIATE HEALTH SERVICE**					
**Absent/inadequate prenatal care**					0.003*
Yes	90	(31.7)	66	(46.5)	
No	194	(68.3)	76	(53.5)	
**Lacking specialized care**					< 0.001*
Yes	43	(15.1)	50	(35.2)	
No	241	(84.9)	69	(64.8)	
**Lacking beds**					0.027*
Yes	27	(9.5)	5	(3.5)	
No	257	(90.5)	137	(96.5)	
**3. DELAY IN RECEIVING CARE AT THE HEALTH UNIT**					
**Delay in diagnosis**					0.477**
Yes	2	(0.7)	2	(1.4)	
No	282	(99.3)	140	(98.6)	
**Delay in case transfer/referral**					0.265*
Yes	8	(2.8)	7	(4.9)	
No	276	(97.2)	135	(95.1)	
**Improper conduct with patient**					0.006**
Yes	1	(0.4)	6	(4.2)	
No	283	(99.6)	136	(95.8)	
**Delay in initiating treatment**					0.489**
Yes	5	(1.8)	4	(1.9)	
No	279	(98.2)	106	(98.1)	

*Pearson’s chi-square test; **Fisher’s exact test

**Table 6:** Logistic regression of the three delays by group, considering combined outcome (near-miss and neonatal deaths). Fortaleza CE, 2017

Variable	Control (95% CI) Gross	Near-miss + Death OR (95% CI) Adjusted
Absent or inadequate prenatal care	**1.9 (1.2–2.8)**	**1.9 (1.2–2.8)**
Lacking beds	**0.4 (0.1–0.9)**	0.4 (0.2–1.1)
Delay due to lack of specialized services	**3.5 (1.9**–**4.9)**	**2.8 (1.7–4.5)**
Improper conduct with the patient	**12.5 (1.6**–**105)**	**10.6 (1.2**–**91.8)**

*OR* Odds ratio; *CI* confidence interval.

## Discussion

The Brazilian Unified Health System (SUS) is public and universal, organized into three levels of care—primary (gateway, where most health problems should be solved), secondary (small hospitals and units with specialist doctors, equipped to provide laboratory imaging diagnostics), and tertiary (hospitals with the capacity to solve complex health problems, generally with surgical wards and intensive care units).

The primary care team is responsible for comprehensive care. The referral between one level and another should comply with agreements between cluster-located clinics forming the Health Care Networks (RAS). The healthcare system was designed in a way to reduce inequalities in access and coverage. Maternal–infant RAS is one of the system’s priorities. However, there are often no available beds to attend specific cases at the tertiary medical facilities [14].

The changes in demographics, economy, and healthcare experienced by Brazil in the last three decades have caused profound changes in different spheres, especially in maternal and child healthcare. SUS enabled the implementation of many programs with a strong potential to reduce maternal and child mortality rates and managed to modify some health indicators in this context. Some public policies have also tried to reduce poverty through conditional cash transfers with the potential to contribute [1].

However, despite the advances, the rates of maternal and perinatal mortality are still high, mostly from causes, considered preventable. We are facing a great challenge as it cannot be guaranteed that the quality of services provided to the mother–child binomial is as planned and that the proposed programs are offered in the necessary quantity [15].

There is a need to identify local factors that persist favoring morbidity and mortality in this period of life, mainly because the Northeast is still one of the Brazilian regions with the highest rate of maternal and child morbidity and mortality.

The results showed that delays in obstetric care increase the risk of neonatal near-miss events and death. The most frequent delays were absent or inadequate prenatal care and delayed access to healthcare due to a lack of specialized service, both classified under the second delay, followed by improper conduct with the patient, classified under the third delay.

A study in eastern Uganda analyzing the three delay model to explain newborn morbimortality reported that the first and third delays contributed to two thirds of the neonatal death outcomes [16]^.^ In another study, the outcome of neonatal mortality was attributed to the third delay [17].

Another study on the same subject that aimed to identify the association between delayed obstetric care and fetal death outcomes reported that the first, second, and third delays contributed to these outcomes [18], showing that delays in maternal care at any levels increase the chances of perinatal complications. We believe that neonatal morbimortality cannot be attributed to a single delay, but to a combination of factors that lead to an unfavorable outcome.

No cases of intrauterine death were evaluated. Cases and controls were selected at childbirth, and only live newborns were included. The first delay (in deciding to seek appropriate medical help, family/patient) was not associated with near-miss or neonatal death. This finding may be explained by the fact that 72.1% of the women were mothers with ≥8 years of education, 82.6% were living with a partner, 84.5% were living in the capital, 70.4% were aged 19–34 years, and 65.4% received an orientation at their place of delivery. This profile may have affected the result that 83.6% of the women did not delay seeking care, 96.5% were aware of the signs of pregnancy, and 87.1% did not refuse care. The prevalence of the first delay was low for all outcomes.

Low maternal education, lack of family support, marital status, maternal age, and lack of confidence about the health service are risk factors for poor maternal and neonatal outcomes [15, 19, 20]. These factors have traditionally been responsible for the worst neonatal outcomes. However, the factors were not significant in our analysis, probably because the population is very homogeneous; all with low socioeconomic status.

Our analysis showed that absent or inadequate prenatal care has a statistically significant association with both near-miss and/or death, with OR values of 1.9. The data showed that 47.2% of the pregnant women in this study started their prenatal care after 12 weeks of pregnancy, 44.8% had less than six consultations, 16.4% had difficulties in prenatal care, and 11.5% were not able to undergo all the recommended tests. These data may be due to some factors already described in the literature. For example, a Brazilian study that evaluated the government database on the National Program for Improving Access and Quality of Primary Care (PMAQ-AB) 2012/2013 showed that pregnant women are accessing and beginning prenatal consultations later than usual, mostly due to a lack of family planning. It is necessary to implement programs focused on educating and helping women identify pregnancy [21].

Despite having reached international indicators and almost equitable levels in all regions, the coverage of prenatal care in Brazil does not guarantee that efficient and high-quality consultations will be provided to these pregnant women; rather, they often show a lack of professional qualifications regarding comprehensive care for the pregnancy and delivery [21, 22]. All of these factors directly affect care conduct, delivery outcomes, and neonatal care, with a direct impact on negative neonatal outcome by increasing NNM and fetal deaths [23]. The quality of prenatal consultations was not assessed. The theoretical framework that we followed to define the first delay evaluates only objective data, such as gestational age at the beginning of care, number of consultations, and performance of laboratory tests recommended by scientific evidence. A prospective study that comes to evaluate the quality of these consultations and the understanding of the information by the patient and her family can bring even more concerning information.

The literature considers a delay in care for pregnant women a determining factor for negative outcomes when they require specialized care [24]. Mother’s origin was statistically significant (*p* = 0.016) on the univariate evaluation. A total of 65 pregnant women came from the countryside; of them, 22 newborns suffered near-miss events and nine died.

There are problems with the availability of hospital beds, a greater concentration of health services in large urban centers, lack of hospital expansion to keep up with population growth, structural and process deficiencies in the referral system, and a counter-referral flow that take these women from one institution to another, culminating in unfavorable maternal outcomes and effects on newborns [25].

Inequity remains the real problem in healthcare access as replicated in some studies [26]. We emphasize here that the region where this study was conducted still has a high rate of maternal and neonatal morbimortality.

Improper conduct with the patient was statistically significant to neonatal near-miss groups (adjusted OR, 12.1) but not neonatal death. When the combined death plus near-miss outcome was evaluated, the OR value was 10.6. The study rates were extremely positive to the institution, reaching 98.4% of proper conduct with the patient after a specialized evaluation.

Our findings show that this is a very relevant issue as reported in a recent systematic review of the third delay, which showed that the third delay significantly contributed to maternal mortality and shed light on the importance of early recognition of maternal and fetal risks. Negative outcomes can occur even under assertive specialized care, but they will be minimized. A large proportion of negative outcomes are preventable [27]. There are multiple and complex reasons for delays involving a lack of supplies, lack of professional technical skills, and bad attitude toward the patient, among others [28, 29]. It is worth mentioning that these data were collected at an undergraduate and graduate teaching maternity center that follows institutional protocols constantly reviewed based on scientific evidence. A good suggestion would be to offer training to all professionals providing maternity and neonatal care on institutional protocols and available scientific evidence, complemented with periodic evaluations of the results after implementing changes.

A study limitation was the possibility of selection bias as the sample was collected from a reference maternity hospital providing high-complexity maternal and neonatal care for the entire state, which enabled the identification of more cases of neonatal near-miss compared to other maternity hospitals, thereby limiting the generalization of our results to medium- and low-complexity maternity hospitals. However, it can be assumed that the results can be extrapolated to several other maternity hospitals in Brazil and other countries that share the same clinical and socioeconomic scenario.

This study is important since it paves the way for new studies in terms of scenarios and other designs. This was the first study developed in the state to evaluate severe neonatal morbidity associated with the three delays, which included maternal reports on difficulties obtaining healthcare, as well as data from medical records throughout pregnancy, delivery, childbirth, and neonatal care. Diversified approach including strategies for improving prenatal care, expanding state hospital structures to reduce transfers to better hospitals, and increasing qualified technical and human resources in emergency care is vital. Collaboration among departments and healthcare service providers is necessary to manage maternal and neonatal care.

## Conclusions

A clear association was found among the occurrence of delays in accessing obstetric care and near-miss events and neonatal death. The most frequent delays were absent or inadequate prenatal care, delay accessing healthcare due to a lack of specialized service, and improper conduct with the patient.

## Data Availability

The datasets used and/or analyzed during the current study are available from the corresponding author upon reasonable request. All co-authors gave full responsibility to the corresponding author to share and/or discuss with editors and reviewers.

## Abbreviations

PMAQ-AB: National program for improving access and quality of primary care
OR: Odds ratio
IC: Confidence interval
NNM: Neonatal near-miss
